# Supplementary material for: One-Step Coating Processed Phototransistors Enabled by Phase Separation of Semiconductor and Dielectric Blend Film
Source: Micromachines (Basel). 2019 Oct 24;10(11):716. doi: 10.3390/mi10110716 (PMC6915368; doi:10.3390/mi10110716)
Supplement: Supplementary file 1 [file micromachines-10-00716-s001.pdf]

One-Step Coating Processed Phototransistors Enabled by Phase Separation of Semiconductor and Dielectric Blend Film

Lin Gao, Sihui Hou, Zijun Wang, Zhan Gao, Xinge Yu and Junsheng Yu

The capacitance of the dielectric layer (C_i) using the two-parallel-plate model:

$$C_i = k\varepsilon_0/d \quad (1)$$

where ε_0 is the vacuum permittivity, k is the dielectric constant of the dielectric, and d is the thickness of the gate dielectric. The thickness of the PMMA film measured by a surface profiler (Contour GK-T) is presented in Figure S1, and the average thickness was about 380 nm.

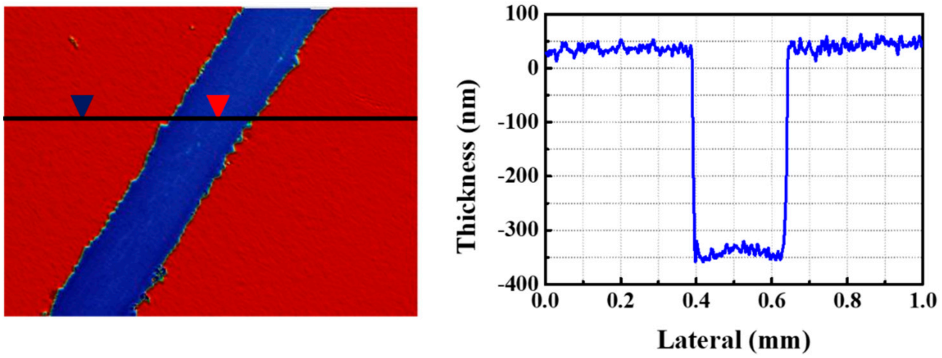

Figure S1. Surface outline with removed part of PMMA and the thickness distribution of PMMA.

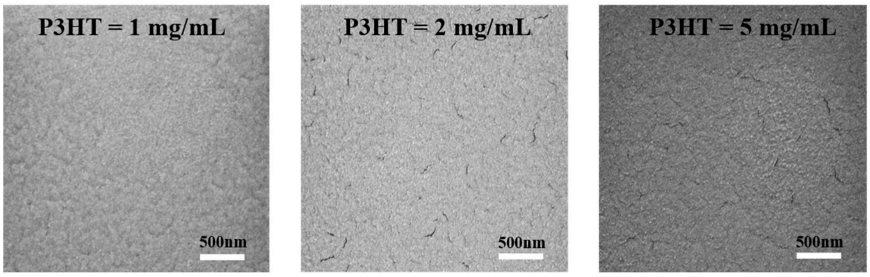

Figure S2. SEM topography images of the films with different P3HT content.
